# A plasmonic gold nanofilm-based microfluidic chip for rapid and inexpensive droplet-based photonic PCR

**DOI:** 10.1038/s41598-021-02535-1

**Published:** 2021-12-02

**Authors:** Abbas Jalili, Maryam Bagheri, Amir Shamloo, Amir Hossein Kazemipour Ashkezari

**Affiliations:** Department of Mechanical Engineering, Sharif University of Technology, Azadi Ave., Tehran, Iran

**Keywords:** Biological techniques, DNA

## Abstract

Polymerase chain reaction (PCR) is a powerful tool for nucleic acid amplification and quantification. However, long thermocycling time is a major limitation of the commercial PCR devices in the point-of-care (POC). Herein, we have developed a rapid droplet-based photonic PCR (dpPCR) system, including a gold (Au) nanofilm-based microfluidic chip and a plasmonic photothermal cycler. The chip is fabricated by adding mineral oil to uncured polydimethylsiloxane (PDMS) to suppress droplet evaporation in PDMS microfluidic chips during PCR thermocycling. A PDMS to gold bonding technique using a double-sided adhesive tape is applied to enhance the bonding strength between the oil-added PDMS and the gold nanofilm. Moreover, the gold nanofilm excited by two light-emitting diodes (LEDs) from the top and bottom sides of the chip provides fast heating of the PCR sample to 230 °C within 100 s. Such a design enables 30 thermal cycles from 60 to 95 °C within 13 min with the average heating and cooling rates of 7.37 ± 0.27 °C/s and 1.91 ± 0.03 °C/s, respectively. The experimental results demonstrate successful PCR amplification of the alcohol oxidase (AOX) gene using the rapid plasmonic photothermal cycler and exhibit the great performance of the microfluidic chip for droplet-based PCR.

## Introduction

Deoxyribonucleic acid (DNA) and ribonucleic acid (RNA) are essential biomarkers for the detection of infectious diseases^1–4^, biosensing^5^, gene expression^6^, and forensic identification^7,8^. Low concentration of these biomolecules in blood and other clinical samples necessitates an amplification process^9,10^. Moreover, pathogens identification take a considerable amount of time; therefore, the sensitive and rapid detection of pathogens plays a vital role in improving human health at the point-of-care (POC) testing^11,12^. Polymerase chain reaction (PCR) has attracted extensive attention in the biological and biomedical fields owing to its ability to amplify and detect the low concentration of nucleic acids^13–15^. Besides, timely diagnosis of infectious diseases such as coronavirus (COVID-19) and human immunodeficiency virus (HIV) requires a rapid PCR method^16–19^. Many attempts to have a fast, efficient, and reliable PCR have been reported in the literature and summarized below.

Recently, many research groups have focused on enhancing the rapidity and quality of the PCR by implementing microfluidic technology^20–24^. This technology possesses numerous advantages, including ease of fabrication, high-speed processing ability, and low reagent consumption^25–27^. However, there are still some major problems with respect to applications of microfluidic systems in biological assays (*e.g.,* PCR) due to several shortcomings of the surface inhibition and contamination^28,29^. In addition, they also face some limitations for simultaneous and multiplex reactions^30^. These drawbacks can be overcome by using the droplets as microreactors. In recent years, droplet-based microfluidic PCR is being widely used in the amplification of nucleic acids^31^. In comparison to typical microfluidic PCR, droplet-based microfluidic PCR benefits from low reagent consumption^32^, low waste production^33^, negligible sample contamination^34^, insignificant surface adsorption^35^, high-speed reactions^36^, and independent monitoring of each droplet^37,38^.

Thermal processing plays a crucial role in developing a rapid, effective, and low-cost PCR system. Since the invention of PCR in 1985 up to now, various thermocycling methods have been performed to improve PCR performance^39–42^. Conventional PCR systems, which utilize a Peltier-based thermal cycler, are capable of dramatically amplifying target nucleic acids. However, they require a long thermocycling time since the heat transfer between the heating block and the plastic PCR tube is slow^43^. Plasmonic photothermal heating of Au-based nanomaterials is one of the most efficient methods to address this limitation^44,45^. In this method, Au-based nanomaterials can be used as photothermal sources upon infrared (IR) laser or light-emitting diode (LED) irradiation. In comparison to a Peltier heating block, plasmonic photothermal heating of Au-based nanomaterials shows higher heating rates and more uniform heating due to the high thermal conductivity of gold^46,47^. Au-based nanomaterials can be mainly classified as Au nanoparticles (AuNPs) and Au nanofilms (AuNFs). Although AuNPs significantly improve the quality of PCR reaction by heat transfer enhancement, they may have an inhibitory effect on nucleic acid amplification at high concentrations^48,49^. Besides, photothermal heating using AuNPs suffers from a nonuniform distribution of nanoparticles^50^. AuNFs may be regarded as a better alternative for nucleic acid amplification than AuNPs. More recently, the photonic thermocycling method, based on the photothermal heating of AuNFs using LED irradiation, has received ever-increasing attention all over the world. The advantages such as low power consumption, rapid temperature ramping capability, and low-cost make this method more beneficial for POC testing^51^.

Polydimethylsiloxane (PDMS) has been widely applied in fabricating microfluidic devices because of its intrinsic advantages, such as biocompatibility, optical transparency, chemical inertness, thermal stability, and low-cost. Although these advantages make it a leading option for PCR applications, the evaporation of samples from PDMS devices during the thermocycling process still remains a tremendous challenge, especially in the case of droplet-based PCR^52–54^. The oleophilic nature of PDMS, accompanied by its porosity, enables mineral oil around the droplets to diffuse into the PDMS structure, resulting in droplets deformation and evaporation during long-term heating^55,56^. In this study, we describe a simple, rapid, inexpensive, and reliable system for droplet-based photonic PCR (dpPCR) analysis by integrating the functions of microdroplet generation, plasmonic photothermal heating of AuNF, and PDMS microfluidic chip. The PDMS surface is modified by adding mineral oil to uncured PDMS to inhibit the evaporation of droplets during PCR thermocycling. Meanwhile, a simple strategy in which a double-sided adhesive tape is used as an interlayer is employed to enhance the bonding strength between PDMS and gold. This inexpensive strategy eliminates the need for complicated protocols and specialized facilities. We present a rapid plasmonic photothermal cycler consisting of a thin Au film and two blue LEDs for fast droplet-based PCR, providing a heating rate of 7.37 ± 0.27 °C/s and a cooling rate of 1.91 ± 0.03 °C/s. According to the rapid temperature ramping rate, 30 thermal cycles from 60 to 95 °C are accomplished within 13 min. Our thermocycler shows a comparable temperature ramping rate to commercial thermocyclers. Finally, the applicability of the proposed device is evaluated by applying it to a PCR assay.

## Material and methods

### AuNF-based microfluidic chip design and fabrication

**Figure 1:**
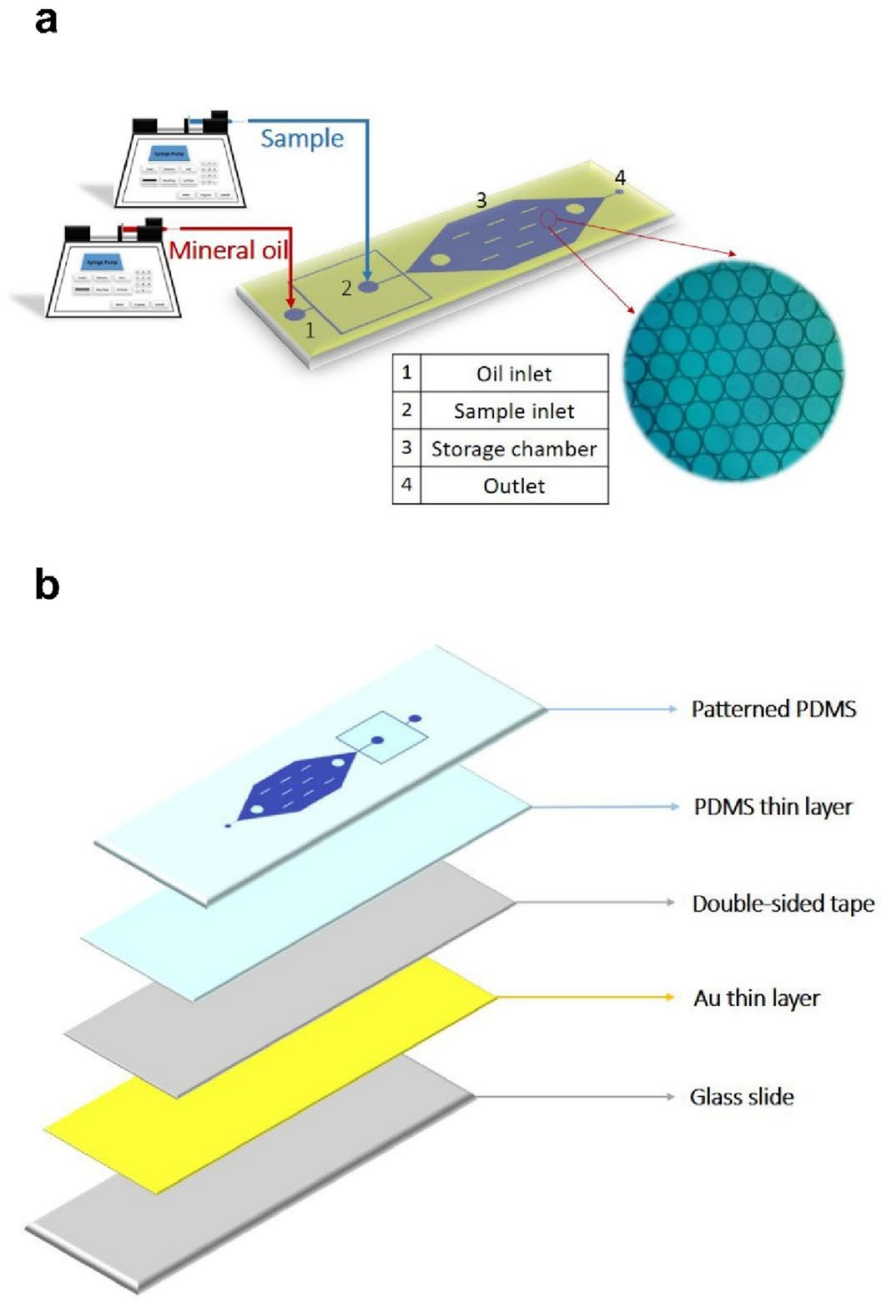
(**a**) Layout of the manufactured microfluidic chip for droplet generation and trapping: two syringe pumps are applied at the inlets, which control sample and oil flow rates to generate monodisperse droplets. (**b**) Illustration of the layered chip structure composed of a patterned PDMS layer, a partially cured PDMS thin layer, a double-sided tape layer, an AuNF layer, and a glass substrate layer.

A schematic of the proposed AuNF-based microfluidic chip is illustrated in Fig. 1a. The device is comprised of two inlet microchannels, a droplet storage chamber, and an outlet microchannel. Two inlet microchannels are joined at a flow-focusing junction that opens to the chamber containing ten supporting pillars. These pillars reduce a large aspect ratio of the chamber, thereby avoiding chamber adhesion to the substrate. The chamber entrance that expands gradually causes droplets to slow down gently, avoiding the coalescence of droplets caused by a collision. The microfluidic chip was fabricated using PDMS by conventional photolithography. The 2D photomask was designed and prepared before fabrication. The preparation of the mold was accomplished to fabricate the chip according to the following procedure. A 2″ silicon wafer was thoroughly cleaned with acetone and isopropyl alcohol. Then the wafer was baked at 95 °C for 7 min to dehydrate the surface. A layer of SU-8 2050 negative photoresist (MicroChem, USA) was spin-coated onto the cleaned silicon wafer to create the flow channels and followed by a soft bake process at 65 °C and then 95 °C for 5 min and 20 min, respectively. The design of the mask was patterned on the wafer by UV exposure with a mask aligner (Azhineh Microsystem, Iran). Then, the mold was hard baked on a hotplate at 65 °C and then 95 °C for 5 min and 10 min, respectively. Development process was accomplished by immersing the substrate into a developer solution (1-methoxy-2-propyl acetate, Sigma Aldrich, USA) for 10 min.

After completion of photolithography, a mixture of PDMS pre-polymer and cross-linker (Sylgard 184, Dow Corning, USA) was prepared at a weight ratio of 10:1, respectively. The mixture was then mixed with light mineral oil (Sigma Aldrich, USA) at various weight percentages. Subsequently, the mixture was degassed in a vacuum desiccator chamber and then poured into the patterned wafer, forming a structured microfluidic substrate. After being partially cured at 70 °C for 40 min, the PDMS was gently peeled off from the mold and then punched with a 1 mm outer diameter disposable biopsy punch (Kai Medical, Japan) to form inlet and outlet holes. The structured microfluidic substrate was bonded to a gold-coated glass microscope slide, which was pre-coated with a thin layer of PDMS, through thermal bonding with a 20 min incubating at 95 °C. To implement the bonding technique, first, a transparent double-sided adhesive tape (Tesa, Germany) with high temperature stability was fixed on the gold-coated glass slide. Degassed uncured PDMS was poured over the double-sided tape and spun at 2000 rpm for 50 s, and later, partially cured at 70 °C on a hotplate for 7 min to provide the thermal bonding. This thin PDMS layer separates the PCR solution from the adhesive tape, avoiding contamination of the sample. Three small pieces of cured PDMS were bonded on the inlets and outlet to interface with tubing. Au nanolayer was deposited by DC magnetron sputtering onto a glass slide. The Au deposition was accomplished at room temperature (25 °C) using a gold target (purity 99.95%) at 37 W with a deposition rate of 4.2 A°/s. All layers of the chip structure are shown in Fig. 1b, and the workflow of the chip fabrication is shown in Supporting Information (Fig. S1).

### Droplet formation

A mixture of mineral oil and appropriate surfactants (3% (v/v) ABIL EM 90 (Goldschmidt GmbH, Germany) and 0.1% (v/v) Triton X-100 (Sigma Aldrich, USA) was used as a continuous phase to avoid the droplet coalescence, and the PCR mixture was used as a dispersed phase. The two immiscible fluids were injected into microchannels through PolyTetraFluoroEthylene (PTFE) tubing attached to 5 ml syringes. The flow rates of the dispersed phase and the continuous phase were controlled by two syringe pumps (FNM Co., Iran) and fixed at 1 µl/min and 3 µl/min, respectively. Highly monodisperse droplets with an average volume of 1.54 nl were generated at the junction and then stored in the chamber. Once the chamber was filled, the inlets and the outlet were completely sealed in order to store the droplets (Fig. 1a). The experimental setup for droplet generation can be found in Supporting Information (Fig. S2).

### PCR condition

To assess the performance of the constructed device applied to dpPCR, amplification of a 325 base pairs (bp) fragment from pPICZ A vector as a template was performed. This vector is a 3.329 kbp yeast expression vector used to express recombinant proteins in Pichia pastoris. Plasmid vectors are extracted by a commercial plasmid DNA extraction kit (FAPDE050, plasmid DNA extraction mini kit, Favorgen, Taiwan). The DNA concentration (${C}_{1}$, ng/μl) was determined by measuring absorbance at 260 nm on a fluorospectrometer (Thermo Fisher, USA), and the measured value was 30 pg/μl. For analysis convenience, the unit of each DNA concentration (ng/μl) was converted into copies/μl. The formula for making this conversion is as follows:

1 $${C}_{2} =({N}_{A} \times {C}_{1} \times {10}^{-9})/(L \times 660)$$

where $L$ is the length of DNA (bp), ${N}_{A}$ is the Avogadro constant which is equal to 6.02 × 10^23^/mol. Moreover, the purity of DNA was also confirmed by the ratio of absorbance at 260 nm and 280 nm. The template was detected using the forward primer of AOX1 (5′-GACTGGTTCCAATTGACAAGC-3′) and the reverse primer of pAOX1 Reverse (5′-GCAAATGGCATTCTGACATCC-3′). The PCR reaction mixture was off-chip prepared by mixing 4 μl of 5× HOT FIREPol^®^ EvaGreen^®^ qPCR Mix Plus (Solis BioDyne, Estonia), 0.5 μl of each forward and reverse primer (10 μM), and 2 μl of DNA template. The total volume of the PCR sample was brought up to 20 μl with sterile double-distilled water. The reaction mixture was injected into the microfluidic chip to generate water-in-oil droplets. Afterwards, the microfluidic chip was placed on the constructed plasmonic photothermal cycler for the amplification process. The thermal cycling protocol included an initial denaturation at 95 °C for 8 min to activate the HOT FIREPol^®^ DNA polymerase, followed by 30 amplification cycles of denaturation at 95 °C for 15 s, annealing at 60 °C for 20 s, extension at 72 °C for 20 s, and then preservation at 10 °C. To evaluate the presence of contamination, no template control (NTC) reaction was conducted using sterile double-distilled water instead of DNA template.

### Theoretical principle of plasmonic photothermal cycling

**Figure 2:**
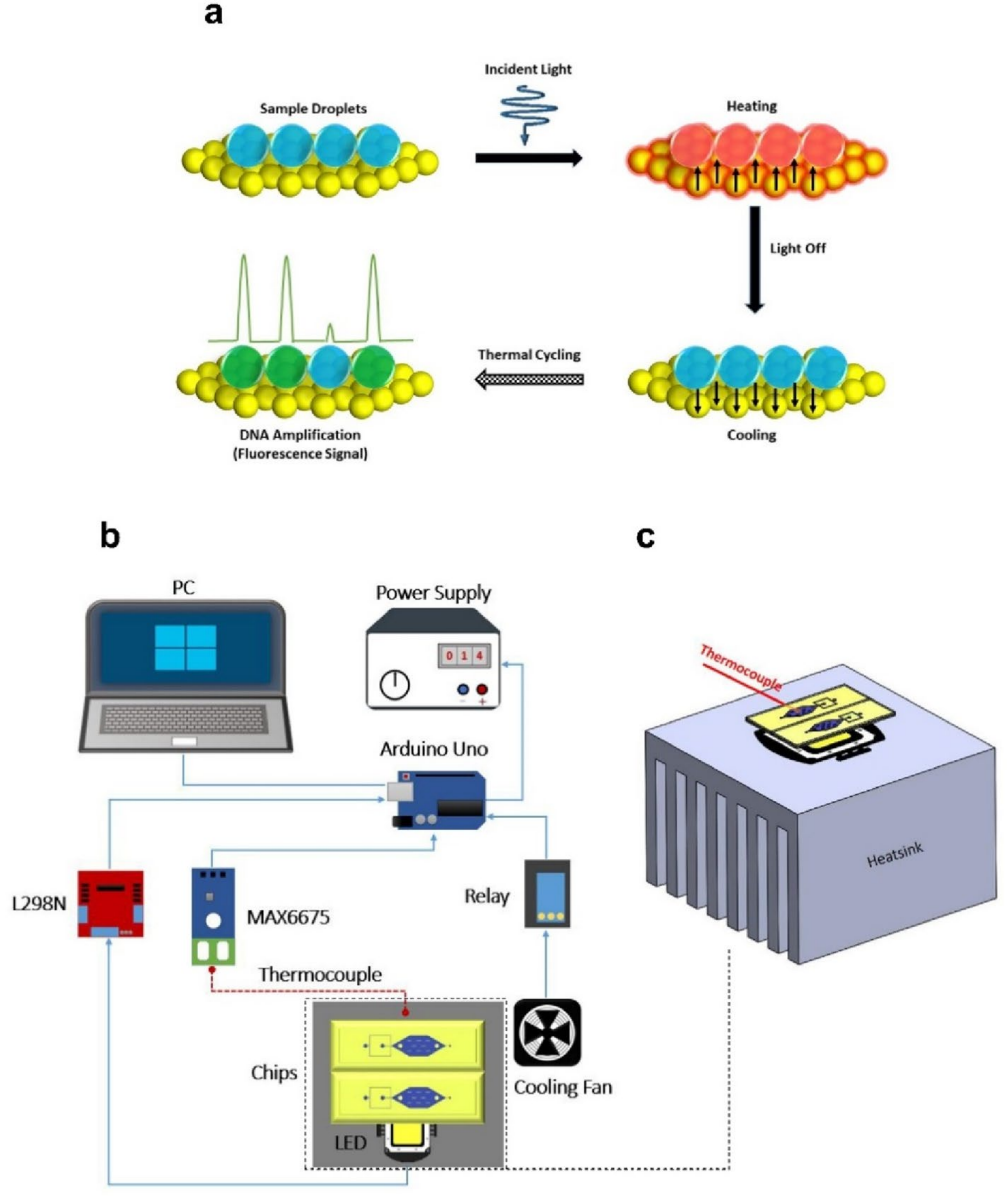
(**a**) Schematic illustration of the fundamental principle of plasmonic photothermal light-to-heat conversion for droplet-based photonic PCR (dpPCR): when a light is turned on, fast heating of sample droplets can be achieved by fast heat diffusion of hot electrons throughout the AuNF. When a light is turned off, the heat dissipation through the AuNF can occur, leading to the cooling of the heated droplets. After thermal cycling, the success of the amplification can be identified by analyzing the fluorescence intensity of droplets. (**b**) Circuit diagram of the plasmonic photothermal cycler. (**c**) An enlarged section of (**b**), indicating the position of the LED, heatsink, chips, and thermocouple.

Figure 2a depicts a schematic of the fundamental principle of plasmonic photothermal light-to-heat conversion for dpPCR. When the incident light with specific wavelengths reaches the surface of the thin Au film, it excites electrons on the surface to higher energy states to become hot electrons with extremely high temperature. Benefitting from the high thermal conductivity of gold (317 W m^−1^ K^−1^) and its plasmon-assisted strong absorption when excited by blue light waves^57^, the heat of these activated electrons can rapidly diffuse throughout the AuNF, resulting in a hot surface with a uniform temperature distribution. Consequently, the surrounding PCR solution temperature can uniformly increase. Once the excitation light is removed, the heated solution can also be cooled owing to the heat dissipation through the AuNF. After completion of the PCR final cycle, the success of positive control (fluorescent droplets) and negative control (non-fluorescent droplets) can be identified by fluorescence signals.

### Plasmonic photothermal cycler

The circuit diagram of the plasmonic photothermal cycler is shown in Fig. 2b,c. The blue SMD LEDs (with a peak wavelength of 450 nm, 10 W at 900 mA injection current) were used for plasmonic heating of the thin Au film. In addition to consuming less power than conventional lasers, LEDs significantly reduce the cost of the device and considerably simplify the configuration. The liquid temperature was adjusted during the thermal cycling process using a closed-loop controller based on a proportional-integral-derivative (PID) feedback control mechanism. The temperature was measured with a type-K thermocouple, and the signals were transmitted to an Arduino Uno microcontroller through a MAX6675 thermocouple module. The thermocouple was placed in the chamber to measure the temperature for closed-loop control. The temperature measurement setup was calibrated with an ice bath and boiling water. Using the configured temperature measuring system, the liquid temperature and time sequences were recorded in real-time for later analysis. To expedite the cooling process, a cooling fan was utilized in the immediate vicinity of the chip. A relay module was used to turn the fan on when the sample temperature was higher than the appropriate temperature for the annealing process. For minimizing the temperature fluctuation around the desired set points, the liquid temperature was continuously controlled by modulating the injection current of the LEDs using an L298N module. Two aluminum (Al) heat sinks were utilized in the system to remove the heat generated from the LEDs since extracting heat from the LEDs enables higher light output and longer life of the device. Thermal cycling using the LEDs and the cooling fan was controlled with the Arduino Uno program. Figure S3 represents the experimental setup for the dpPCR, along with the execution profile of the thermal cycler.

### Image acquisition and analysis

An inverted fluorescence microscope (TCM400, Labomed Co., USA) equipped with a digital camera and a 4× objective lens was used to record the droplet generation process (see Movie S1). After the thermal cycling process, microdroplets were transferred into an observable chamber via PTFE tubing. A 10× objective lens was used to capture the fluorescence images with a higher resolution. Additionally, the excitation light and the EvaGreen emission were filtered by a B-excitation filter. By utilizing the EvaGreen dye-based Master Mix, the PCR amplification was analyzed by fluorescence intensity of droplets instead of gel electrophoresis. An image analysis software (ImageJ, USA) was used to detect the fluorescence intensity of the droplets in the positive and negative controls, and the results were analyzed using the equation of Poisson distribution:

2 $${P}_{k} = \frac{{\lambda }^{k}{ e}^{-k}}{k!}$$

where ${P}_{k}$ is the probability of encapsulating k DNA molecules in one droplet, and $\lambda$ refers to the average number of DNA molecules per droplet which could be calculated using the concentration of stock DNA template (${C}_{2}$) obtained from Eq. (1), mean volume of droplets (${V}_{d}$), and a dimensionless number representing the dilution factor ($D$):

3 $$\lambda ={DC}_{2}{V}_{d}$$

## Results and discussion

### Performance of the PDMS microfluidic chip

**Figure 3:**
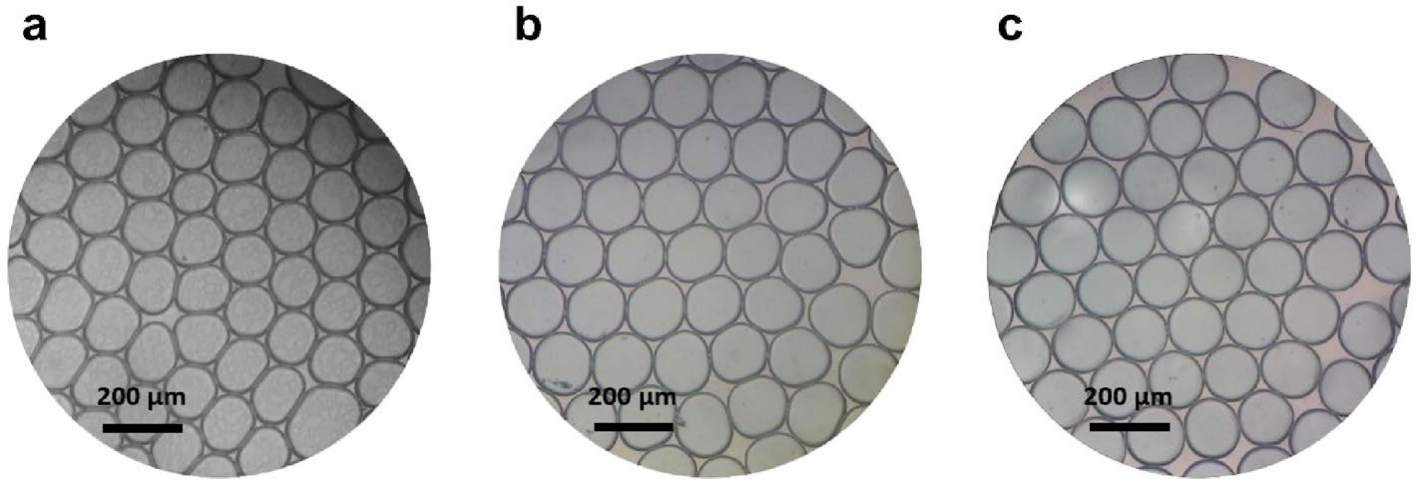
Comparison of droplets behavior in oil-blended PDMS chip with (**a**) 0, (**b**) 3, and (**c**) 7% mineral oil after amplification (bright-field microscopy images).

Our droplet-based PCR workflow comprises three key steps. In the first step, the oil-blended PDMS chip was fabricated. PDMS is commonly used in biochip fabrication owing to its biocompatibility, excellent optical transparency, and heat stability. Due to its highly porous structure, sample evaporation during long-term thermal cycling remains a major restriction of PDMS-based chips in micro PCR, and thus the working performance of these devices may be affected. This is especially a matter of considerable concern when the nanoliter droplets and high temperatures are involved, as is found in this study. PDMS is prone to adsorption of mineral oil around the droplets over time, resulting in droplets evaporation and deformation under many times of thermal cycling. Thus, we used a strategy from the earlier study reported by Bian et al.^58^, using an oil-saturated PDMS chip to suppress droplet evaporation. We added mineral oil to uncured PDMS to reduce unfavorable adsorption of mineral oil onto the PDMS walls. Moreover, to investigate the impact of mineral oil on the protection of water droplets from evaporation during thermal cycling, oil-blended PDMS chips at three mineral oil weight percentages of 0, 3, and 7% were fabricated. Figure 3 shows bright-field microscopy images of the droplets in three mineral oil-blended PDMS chips with different concentrations of mineral oil after the thermal cycling process. It can be seen that the PDMS chip containing 0% mineral oil was unable to maintain the stability of the droplets, which indicates that pure PDMS suffers from a serious deficiency in PCR amplification. Additionally, this problem was not completely solved by adding 3% mineral oil to the PDMS, while the PDMS chip containing 7% mineral oil exhibited only slight changes in droplet volume. These results demonstrate that blending a sufficient amount of mineral oil into the PDMS is a reliable surface modification process, which effectively prohibits mineral oil from being adsorbed by oleophilic PDMS. This method protects droplets surrounded by mineral oil from evaporation and deformation during thermal cycling. Moreover, this technique does not require special surface treatment equipment and thus simplify the surface modification process. This method is particularly noteworthy in resource-limited circumstances.

In the second step, stable and monodisperse droplets with a mean volume of 1.54 nl were generated by introducing the sample into the central channel and the surfactant-stabilized mineral oil into the side channels. It is worth emphasizing that the oil and its stabilizing surfactants play a crucial role in the stability and biocompatibility of the droplets, which are essential for the success of droplet-based PCR amplification. In this study, thermally stable mineral oil and surfactants (0.1% Triton X-100 and 3% ABIL EM 90) were used for the generation of stable droplets due to their biocompatibility. After filling the chamber, the inlets and outlet were completely sealed in order to on-chip thermal cycling. The reaction chamber can be filled with droplets in less than 5 min.

In the third step, on-chip PCR amplification using the plasmonic photothermal cycler was conducted. It is clear that the transmittance of the Au-coated glass slide decreases with an increase in the thickness of the gold nanolayer. In order to end-point fluorescence readout via fluorescence microscopy, it seems necessary to transfer the droplets from the AuNF-based microfluidic chip into the observable chamber immediately after amplification. After transferring the emulsion to the observation chamber, the fluorescence signals of the droplets were observed with the inverted fluorescence microscope. Finally, data analysis was carried out with ImageJ software.

### Performance of the plasmonic photothermal cycler

**Figure 4:**
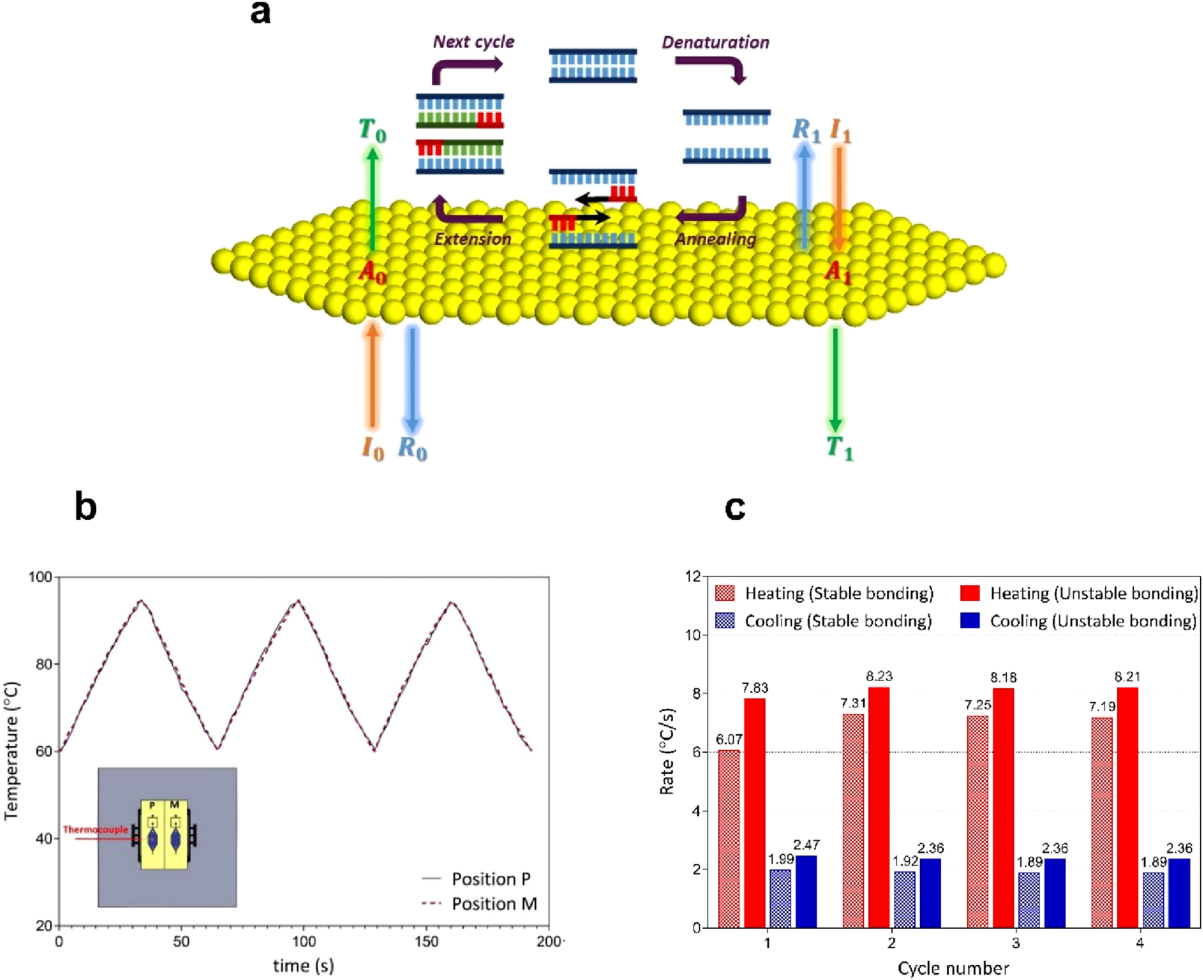
(**a**) Schematic depiction of the photonic PCR thermal cycling, consisting of three successive temperatures for denaturation, annealing, and extension, using the Au nanofilm (AuNF) excited by the LEDs. Light that encounters AuNF (I_0_ and I_1_) can be reflected (R_0_ and R_1_), transmitted (T_0_ and T_1_), or absorbed (A_0_ and A_1_). (**b**) Comparison of temperature profiles of the parallel reaction chamber (PRC) between position P and position M to ensure that the temperature ramping rate of both chambers is the same. Inset shows a schematic of positions P and M. (**c**) Comparison of heating and cooling rates of the sample using the constructed chip with (stable bonding) and without (unstable bonding) tape layer.

As illustrated in Fig. 4a, emitted light (I_0_ and I_1_) that encounters an AuNF can be reflected (R_0_ and R_1_), transmitted (T_0_ and T_1_), or absorbed (A_0_ and A_1_). Only the absorbed light can be converted into heat, leading to an increase in the PCR sample temperature. The use of AuNF with a 120 ± 5 nm thickness can provide high optical absorption, as well as produce the most heat due to its maximum light-to-heat conversion efficiency, as evaluated previously^57^. Additionally, the absorption coefficient is a function of color due to the fact that materials often absorb some colors better than others. The thin Au film exhibits the highest light-to-heat conversion efficiency for the blue LED thanks to the strong optical absorbance at 450 nm wavelength. Consequently, a 120 ± 5 nm AuNF deposited on a glass slide was served as a light-to-heat converter, and two blue LEDs with a wavelength of 450 nm placed on the top and bottom sides of the AuNF-based chip were used as a heating source to provide thermal cycling for PCR.

The temperature of the sample was measured by the type-K thermocouple. The thermocouple was located inside a parallel reaction chamber (PRC) instead of the main reaction chamber (MRC), thereby minimizing any contamination of the sample. Both chambers were placed at an equal distance from the cooling fan. To ensure the sameness of the temperature ramping rate of PRC (at position P) and MRC (at position M), the PRC was located at both positions, and the thermocycling process was performed. The temperature profiles of the PRC at position P and position M are shown in Fig. 4b. It can be found that no significant temperature difference was observed in the temperature profiles at both positions since all conditions were the same. Thus, the temperatures for the thermal cycling process were measured by using the thermocouple inside the PRC for subsequent experiments.

An issue of concern with the use of PDMS is the weak bonding of such a polymer to gold substrates owing to the low energy of the PDMS surface. This is especially true for the microchips with high operating temperature values such as PCR chips in which solution leakage can cause a serious problem. At first, we tried to accomplish the PCR amplification using the chip created by the conventional thermal bonding. The bond strength between the PDMS thin layer and the gold-coated glass was insufficient to endure the high temperatures required for PCR assay. As a result, the failure in the PCR process occurred after 4 cycles due to the detachment of the PDMS from the gold surface. To address this issue, a tape-assisted thermal bonding strategy in which a thin double-sided adhesive tape was used as a connection layer between the gold-coated glass slide and the PDMS thin layer was employed to fabricate the PCR chip. Eventually, by using this method, the PCR amplification was completely performed. Figure 4c shows a comparison of heating and cooling rates of the sample for only 4 thermal cycles using the constructed chip with (stable bonding) and without (unstable bonding) adhesive tape layer. Although the chip without tape showed a relatively high temperature ramping rate (average heating rate of 8.11 °C/s and cooling rate of 2.38 °C/s for the first 4 thermal cycles), the weakness of the bonding strategy without tape prevented the PCR process from continuing for more cycles. Therefore, we utilized double-sided tape to have a complete PCR process, benefiting from rapid plasmonic photothermal cycling (average heating rate of 6.96 °C/s and cooling rate of 1.92 °C/s for the first 4 thermal cycles).

**Figure 5:**
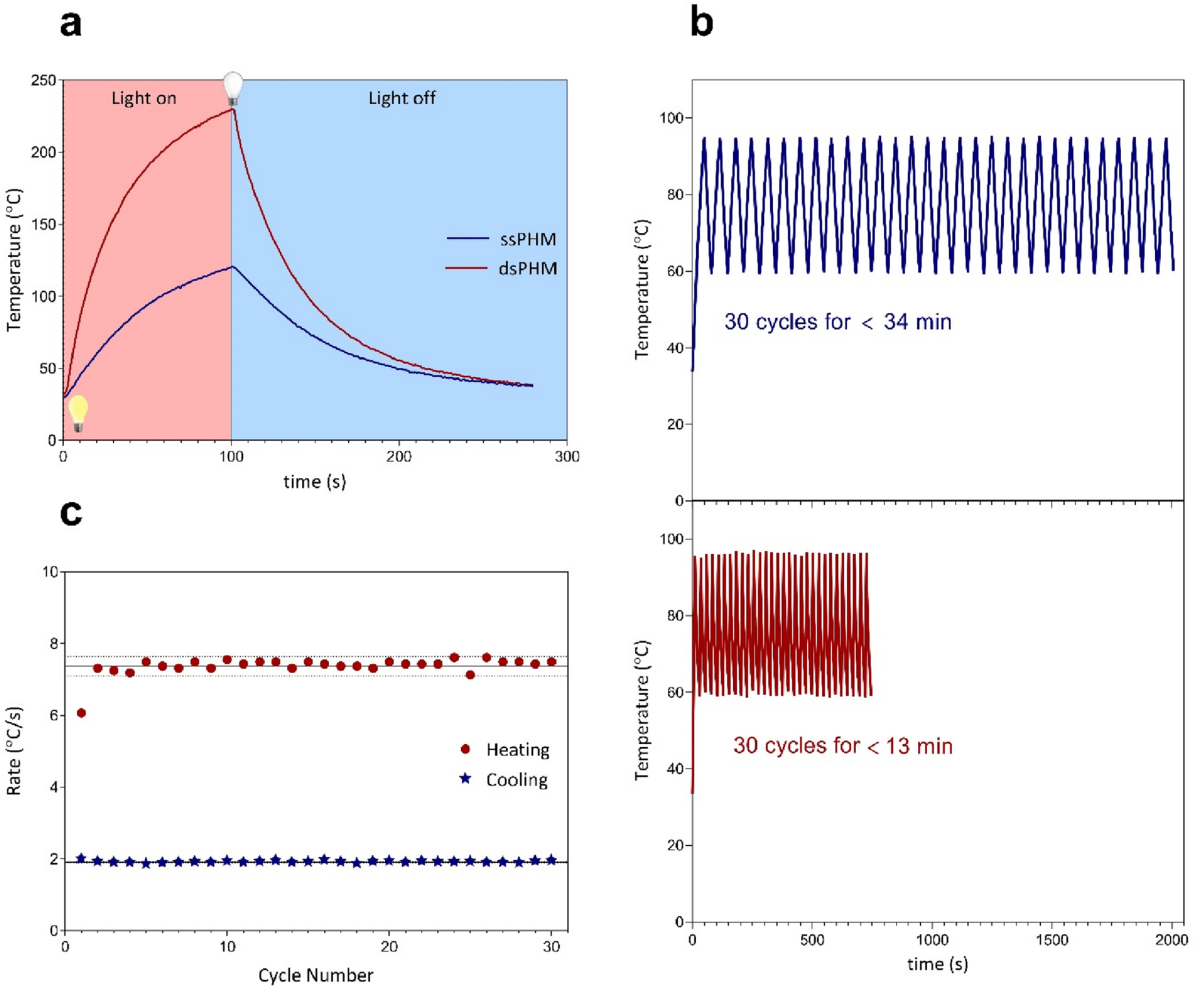
(**a**) Temperature profiles of the single-sided plasmonic heating mechanism (ssPHM) and the dual-sided plasmonic heating mechanism (dsPHM) for the blue light. (**b**) Temperature profiles of the sample in the ssPHM and the dsPHM for denaturation-to-annealing ramp and vice versa (30 PCR thermal cycles, 95 °C for 0 s and 60 °C for 1 s). (**c**) Heating and cooling rates obtained from the dsPHM thermal cycling (average heating and cooling rates of 7.37 ± 0.27 °C/s and 1.91 ± 0.03 °C/s, respectively). Solid lines indicate average values. Dashed lines are placed one standard deviation away from averages.

To investigate the effect of a single-sided plasmonic heating mechanism (ssPHM) and a dual-sided plasmonic heating mechanism (dsPHM) on the heating rate, two PCR experiments were performed. Firstly, the thin Au film was irradiated using a LED from the bottom side. Subsequently, the AuNF was simultaneously illuminated from the top and bottom sides by using the dsPHM to achieve a higher temperature ramping rate. As it can be seen in Fig. 5a, the ssPHM showed a temperature increase up to 120 °C under excitation of the blue light for 100 s, whereas the dsPHM exhibited a rapid temperature increase up to 230 °C. Figure 5b shows the temperature profiles of the sample in the ssPHM and the dsPHM for denaturation-to-annealing ramp and vice versa (30 PCR thermal cycles, 95 °C for 0 s and 60 °C for 1 s). This result showed that the 30 thermal cycles were performed within 34 min and 13 min for the ssPHM and the dsPHM, respectively. Based on these results, the ssPHM was still relatively time-consuming compared to the dsPHM; therefore, the dsPHM was used for subsequent experiments due to its capability of reducing the PCR amplification time. According to the thermal cycling results, the average heating and cooling rates were calculated by dividing the temperature difference between successive maximum and minimum temperatures by the time interval between them until no reduction was observed in the intensity of light. As shown in Fig. 5c, the average heating and cooling rates of the dsPHM at the control points are 7.37 ± 0.27 °C/s and 1.91 ± 0.03 °C/s, respectively.

**Figure 6:**
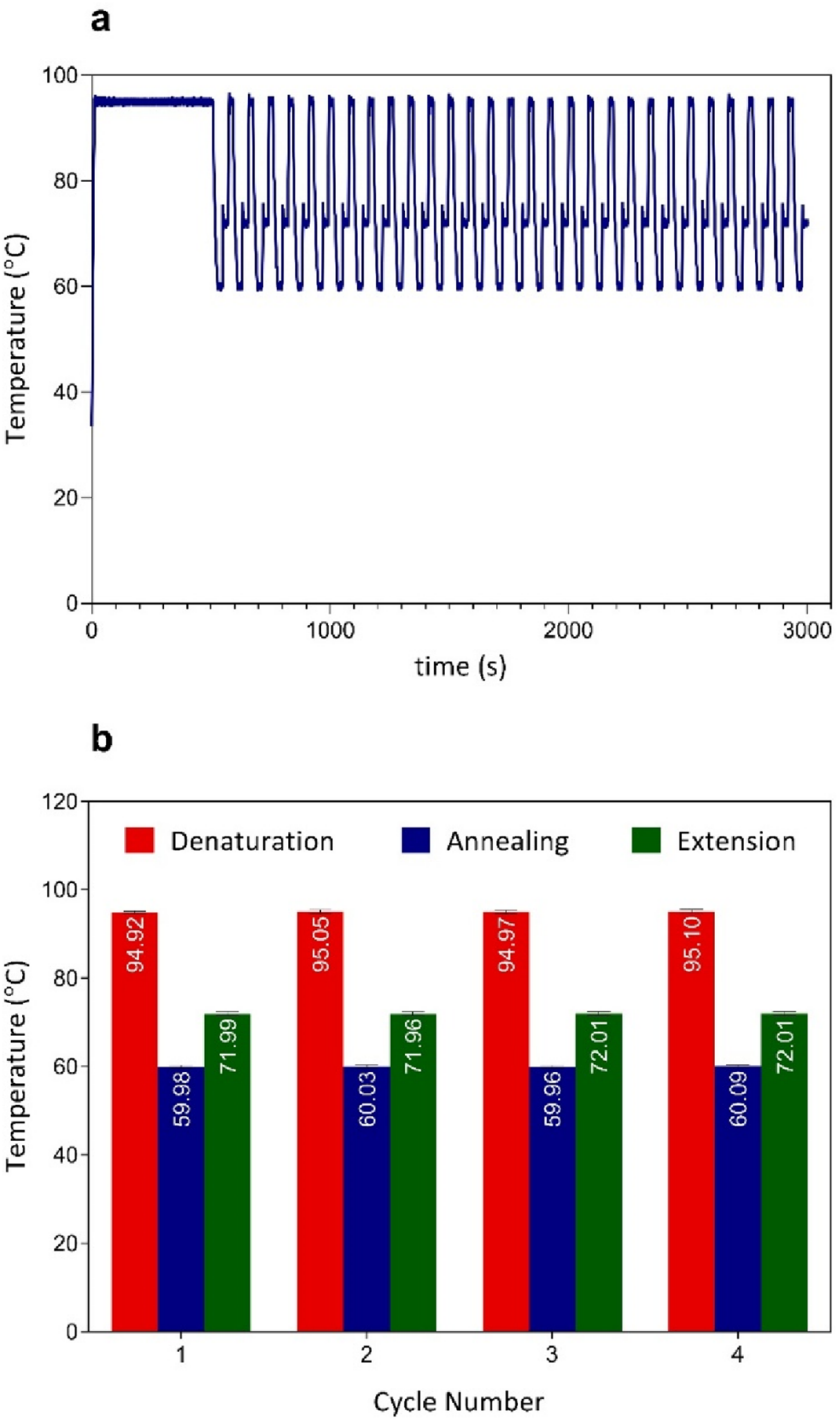
Illustration of the plasmonic photothermal cycling results with the dsPHM. (**a**) PCR thermal profile of a complete PCR reaction. The thermocycling program settings are 95 °C pre-denaturation for 8 min followed by 30 cycles of 95 °C (denaturation), 60 °C (annealing), and 72 °C (extension) for 15 s, 20 s, and 20 s, respectively. (**b**) Evaluation of temperature accuracy and stability. The average values with standard deviation at 95 °C, 60 °C and 72 °C are 94.99 ± 0.41 °C, 60.02 ± 0.37 °C and 72.01 ± 0.39 °C, respectively.

To indicate the feasibility of performing droplet-based PCR on the chip using the plasmonic photothermal cycler, a complete PCR experiment was accomplished by the constructed platform (Fig. 6a). The thermal cycling protocol included a pre-denaturation at 95 °C for 8 min, followed by 30 amplification cycles (denaturation at 95 °C for 15 s, annealing at 60 °C for 20 s, and extension at 72 °C for 20 s). To assess the functionality of the plasmonic photothermal cycler, a PCR experiment was performed using a commercial PCR thermal cycler (Mic qPCR cycler, Bio Molecular Systems, Australia). Comparing to the total processing time of the commercial thermal cycler (75 min), the presented platform (50 min) showed a significant improvement. The temperature fluctuation around the desired set points was reduced by experimentally optimizing the operational variables of the PID controller. As shown in Fig. 6b, the average temperature values with standard deviation at each temperature set point for denaturation, annealing, and extension were calculated as 94.99 ± 0.41 °C, 60.02 ± 0.37 °C, and 72.01 ± 0.39 °C, respectively, showing excellent temperature stability. The temperature accuracy is comparable to commercially available thermocyclers (e.g., T100, Bio-Rad, USA) with an accuracy of ± 0.5 °C. These results demonstrate the ability of the plasmonic photothermal cycler to carry out a rapid and precise PCR thermal cycling.

### Rapid droplet-based PCR amplification and end-point fluorescence detection

**Figure 7:**
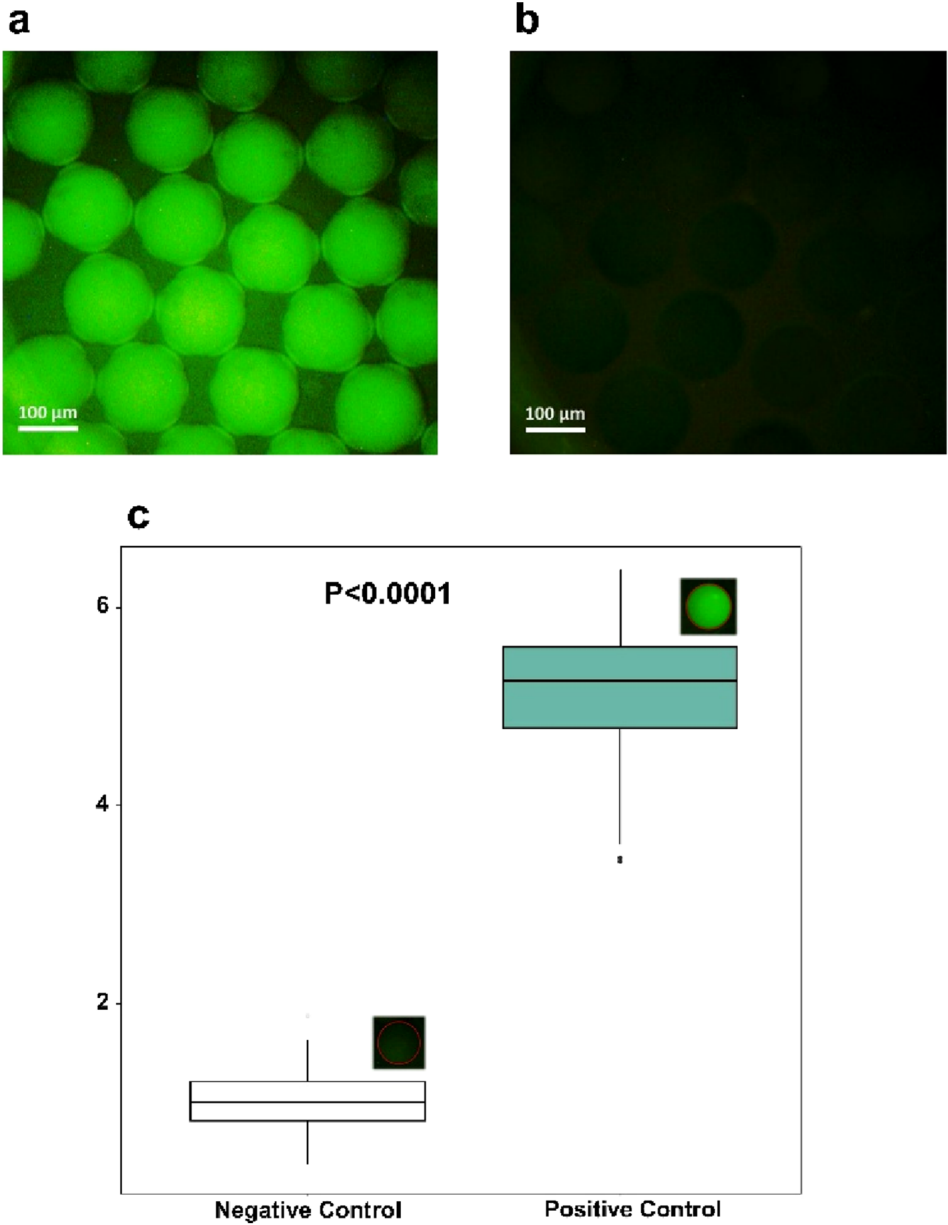
Representative fluorescence microscopy images of the droplets after 30 thermal cycles for (**a**) positive control reaction and (**b**) no template control (NTC) reaction. (**c**) Normalized fluorescence intensity of the positive (right) and negative (left) end-point signal. The results of the positive droplets were normalized to that of the NTC droplets. The average green pixel intensity of the positive droplets was significantly higher than that of the NTC ones. The bottom and top hinges of the boxplot show the first and third quartiles of a sample of 300 randomly selected droplets represented in each box, respectively. The medians are depicted by the horizontal lines in the middle, and the whiskers indicate min and max values. Outliers and the p-value are also shown. The p-value is calculated using two-tailed t-test for independent samples.

To evaluate the functionality of our rapid dpPCR system, a positive control reaction containing the AOX gene as a template and a no template control reaction were performed. To eliminate the need for gel electrophoresis or other post-amplification processes, EvaGreen dye-based Master Mix was used. End-point fluorescence detection was achieved using an inverted fluorescence microscope. Fluorescence images of the positive and negative reactions are shown in Fig. 7a,b. The green fluorescence image of the droplets in the positive control reveals that the target DNA was successfully amplified using our rapid photonic PCR system. No positive signal and no contamination were observed when negative control was conducted using sterile double-distilled water instead of the template. As shown in Fig. 7c, the average fluorescence intensity of the positive droplets was normalized to that of the NTC droplets. The average green pixel intensity of the positive droplets (right) was significantly higher (approximately 5 times) than that of the NTC ones (left). As mentioned previously, a 325 bp DNA fragment was chosen as the target sequence. It should be noted that the performance of such droplet-based assays is detrimentally affected if longer amplicon lengths are designated^59^. In this regard, Fig. S4 shows the significant decrease in the fluorescent intensity of positive control droplets when appropriate PCR reagents with comparable template concentration are used to amplify a DNA fragment of length 1800 bp.

The successful application of EvaGreen intercalating dye in droplet-based PCR relies on the specific amplification of DNA targets. Typically, EvaGreen can bind to double-stranded DNA (dsDNA) and single-stranded primer sequences; therefore, primer-dimer or other nonspecific DNA fragments can contribute to the fluorescence emission of negative droplets. To verify the amplification of the correct product, a PCR melting curve analysis was performed by the Mic qPCR cycler. As it can be found in Fig. S5, a single peak at 86.05 °C was observed for the amplified DNA in the melt curve. This revealed the specific amplification without the appearance of nonspecific PCR products, which was due to the design of specific primers.

### Quantitative analysis with dpPCR

**Figure 8:**
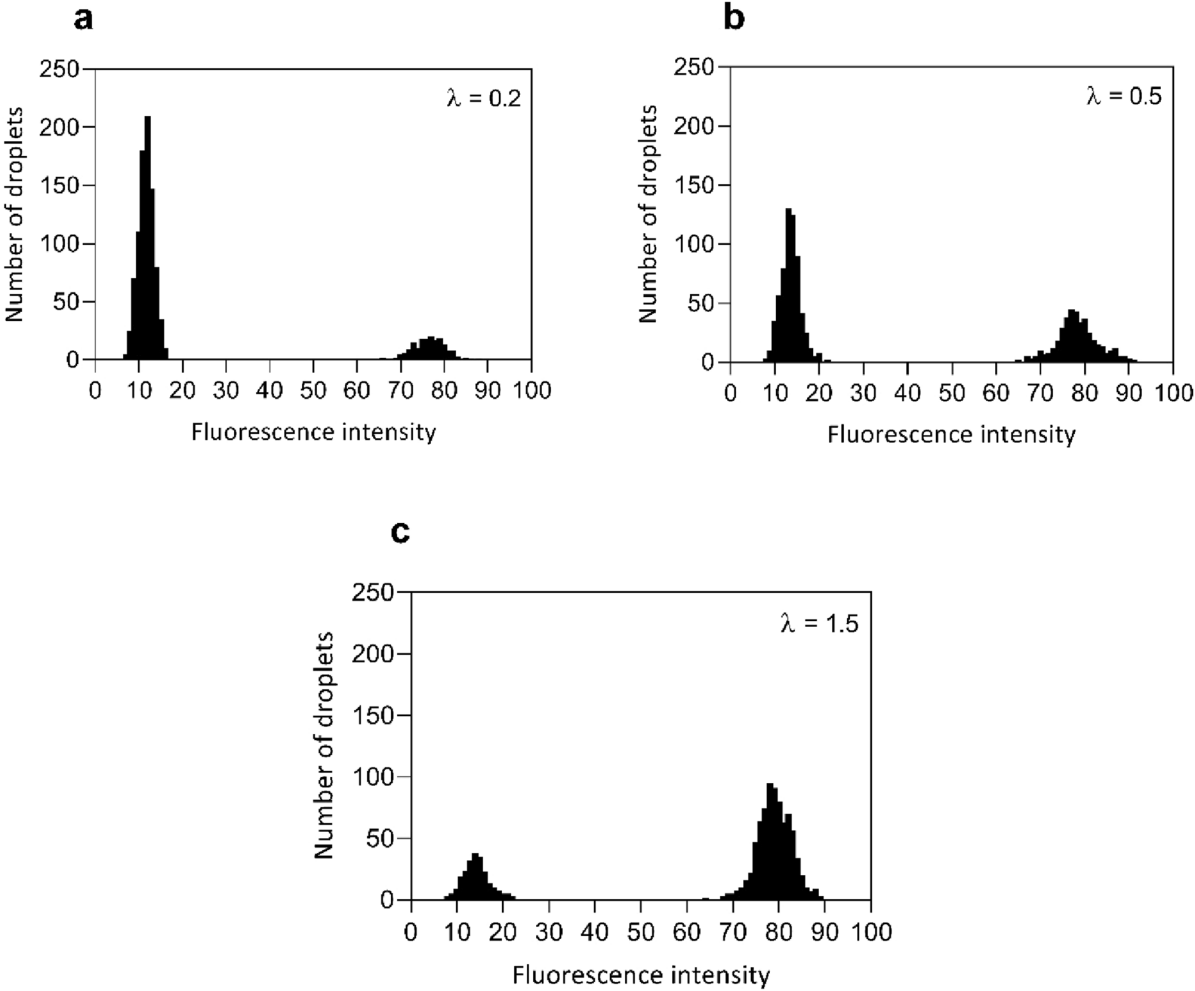
Histograms of fluorescence intensity values for over 1000 droplets at (**a**) λ = 0.2, (**b**) λ = 0.5, and (**c**) λ = 1.5.

Statistical analysis of droplets resulting from emulsion PCR with different dilutions of DNA templates was performed to quantify the performance of our dpPCR system. Over a thousand droplets were randomly selected after the amplification to measure their fluorescence intensities. Figure 8a–c show the distribution of the fluorescence intensity values of the analyzed droplets resulting from PCR mixtures with varying DNA concentrations [i.e., λ = 0.2, 0.5, and 1.5 calculated from Eq. (3)]. The fluorescence intensity values in the droplets containing target DNA increased significantly compared with the droplets containing no target DNA. To show the statistically significant difference between negative and positive droplets, the p-values are calculated to be less than 0.0001 for all three experiments using the two-tailed t-test for independent samples. It can be seen that the fraction of positive droplets decreased as the concentration of the DNA template in the samples was diluted. The fractions of the resultant fluorescent-positive droplets were 17.15%, 38.8%, and 77.15% when the diluted DNA template was used at λ = 0.2, 0.5, and 1.5, respectively. These results agree closely with the theoretically expected Poisson distributions for the particular λ values used (i.e., 18.1%, 39.4%, and 77.7% at λ = 0.2, 0.5, and 1.5, respectively). This shows that the proposed dpPCR system is an accurate and reliable tool to quantify DNA molecules.

## Conclusion

In our previous studies, we designed different types of microfluidic devices that could be implemented for numerous applications^60–66^. In this work, we proposed a gold nanofilm-based microfluidic chip integrated with a rapid plasmonic photothermal cycler for droplet-based photonic PCR. The monodisperse droplets which were generated using flow-focusing geometry and then accumulated in the chamber served as microreactors for on-chip amplification. Adding a sufficient amount of mineral oil into the PDMS protected the droplets from evaporation during long-time thermal cycling. Moreover, the use of double-sided adhesive tape could overcome the problem of bonding weakness between the PDMS thin layer and the gold-coated glass slide. This tape-assisted thermal bonding strategy is suitable for metal (*e.g.,* gold)-based chips fabricated using PDMS, even those face high temperature values during their operation. This is an inexpensive, simple, and efficient strategy that requires no complicated protocols and specialized facilities. The plasmonic photothermal cycler in which the gold nanofilm was excited by two LEDs from the top and bottom sides provided the average heating and cooling rates of 7.37 ± 0.27 °C/s and 1.91 ± 0.03 °C/s, respectively. Such a system enabled rapid thermal cycling from 60 °C (annealing) to 95 °C (denaturation) within 13 min for 30 cycles. The complete PCR thermal cycling results indicate the maximum temperature deviation of ± 0.41 °C at the set points, showing comparable temperature accuracy to commercial thermocyclers. Attributed to the rapid and accurate thermal cycling, on-chip PCR amplification of the alcohol oxidase gene was successfully demonstrated. In the future, we will try to make microdroplets formation more automated to improve the portability of this system. Our dpPCR system offers remarkable features, including (1) equipment-free and efficient modification of PDMS surfaces for reduction of water loss; (2) simple, cost-effective, and reliable PDMS to gold bonding strategy; (3) affordable and rapid plasmonic photothermal cycler; (4) low power consumption; (5) simple and compact configuration. According to these prominent advantages, we believe that our platform is quite helpful for a broad range of applications, such as molecular diagnostics.

## Supplementary Information

Supplementary Figures.

Supplementary Movie S1.
