# Supplementary material for: A plasmonic gold nanofilm-based microfluidic chip for rapid and inexpensive droplet-based photonic PCR
Source: Sci Rep. 2021 Dec 2;11:23338. doi: 10.1038/s41598-021-02535-1 (PMC8639772; doi:10.1038/s41598-021-02535-1)
Supplement: Supplementary file 1 — Supplementary Figures. [file 41598_2021_2535_MOESM1_ESM.docx]

**Supplementary information**

**A plasmonic gold nanofilm-based microfluidic chip for rapid and inexpensive droplet-based photonic PCR**

Abbas Jalili^1,†^, Maryam Bagheri^1,†^, Amir Shamloo^1,*^ , Amir Hossein Kazemipour Ashkezari^1^

^1^ Department of Mechanical Engineering, Sharif University of Technology, Tehran, Iran

^†^ These authors contributed equally to this work

^*^ Corresponding author: Dr. A. Shamloo, Department of Mechanical Engineering, Sharif University of Technology, Azadi Ave., Tehran, Iran, Email: [shamloo@sharif.edu](mailto:shamloo@sharif.edu)

Tel: 98-21-66165691, Fax: 98-21-66165599


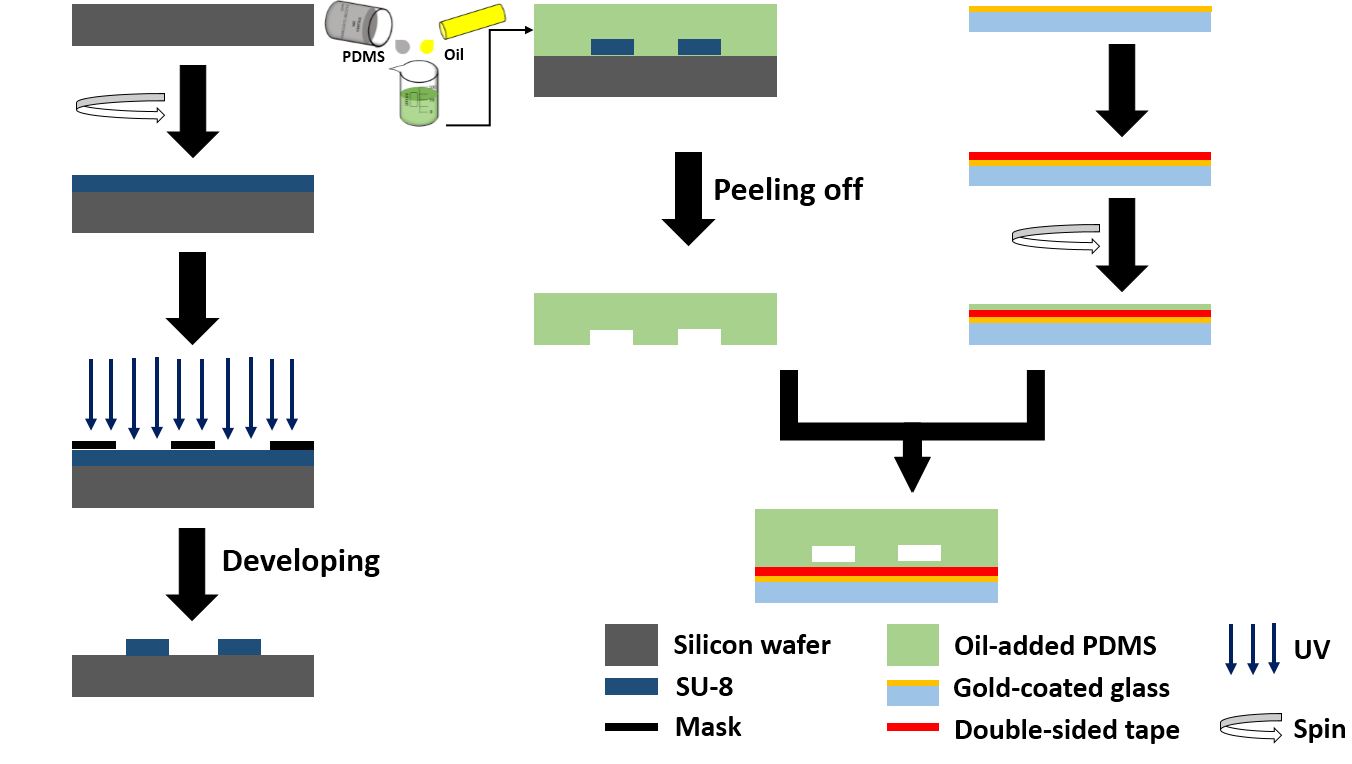
Fig. S1. Workflow of the chip fabrication, including photolithography and tape-assisted thermal bonding.


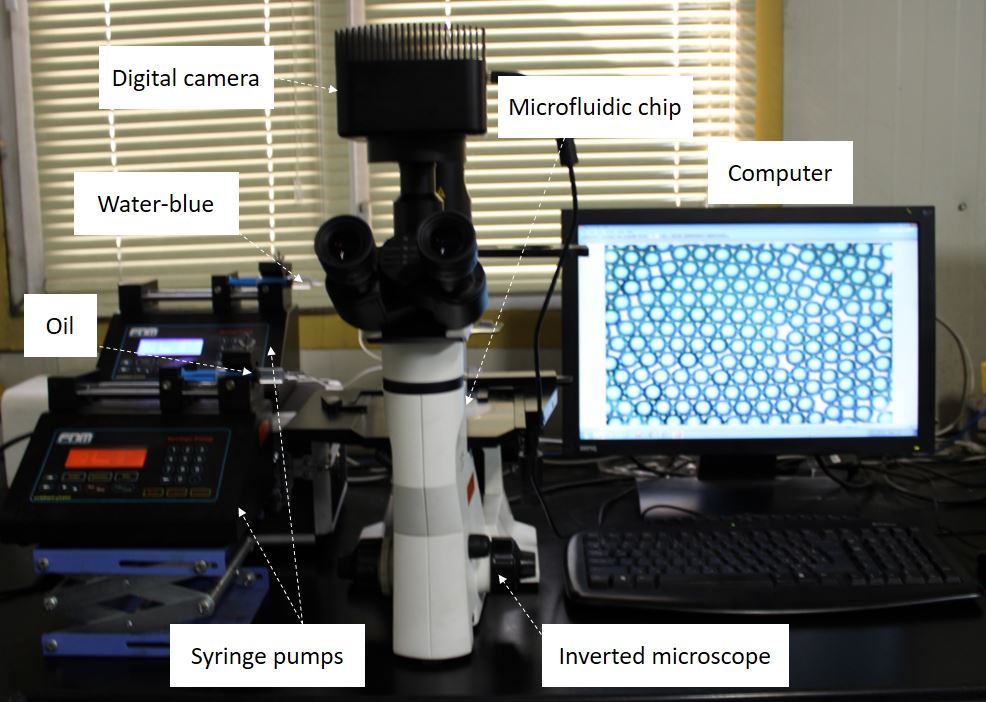


Fig. S2. Photograph of the experimental setup for droplet generation.


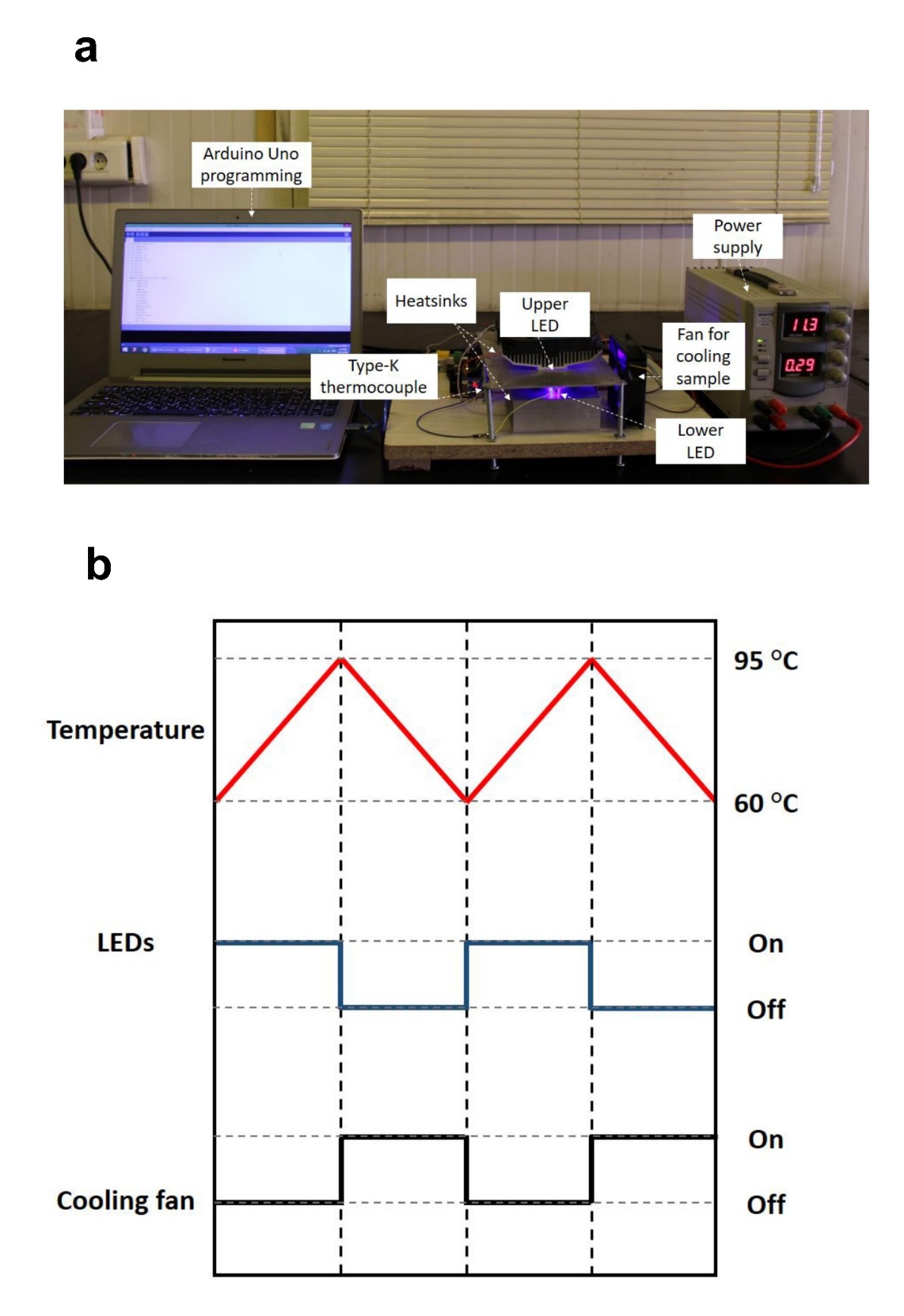


Fig. S3. (a) The experimental setup used for the droplet-based photonic PCR (dpPCR). (b) Execution profile of the plasmonic photothermal cycler.


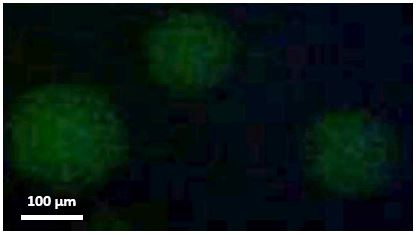


Fig. S4. The fluorescence image of EvaGreen dye after complete thermal cycling of a 1800 bp gene fragment.


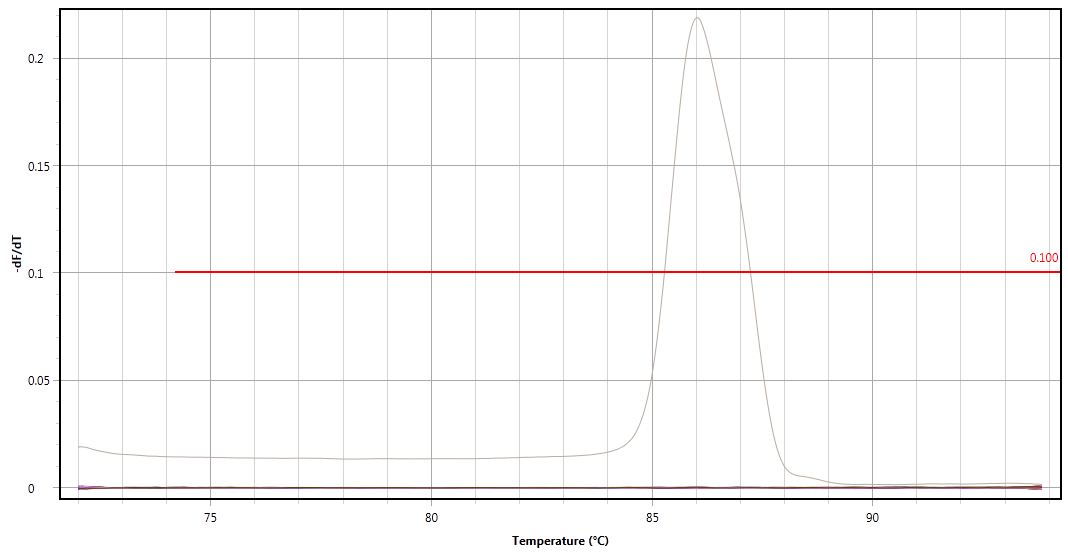
Fig. S5. Specificity of the PCR assay with fluorescence melting curve for a 325 bp fragment from pPICZA vector performed with a Mic qPCR cycler, melting temperature: 86.05 °C.

**Description of movie for droplet generation**

**Movie S1** Displays the generation of the water-in-oil droplets.
